# Population pharmacokinetics and individualized dosing of tigecycline for critically ill patients: a prospective study with intensive sampling

**DOI:** 10.3389/fphar.2024.1342947

**Published:** 2024-01-29

**Authors:** Wei Su, Shuping Song, Jieqiong Liu, Haitao Yu, Binbin Feng, Yinshan Wu, Feng Guo, Zhenwei Yu

**Affiliations:** ^1^ Intensive Care Unit, Sir Run Run Shaw Hospital, School of Medicine, Zhejiang University, Hangzhou, China; ^2^ Department of Pharmacy, The 903rd Hospital of PLA Joint Logistic Support Force, Hangzhou, China; ^3^ Department of Clinical Laboratory, Sir Run Run Shaw Hospital, School of Medicine, Zhejiang University, Hangzhou, China; ^4^ Department of Pharmacy, Sir Run Run Shaw Hospital, School of Medicine, Zhejiang University, Hangzhou, China

**Keywords:** tigecycline, population pharmacokinetics, critically ill, creatine clearance, individualized dosing

## Abstract

**Background:** Due to the heterogeneity of critically ill patients, the pharmacokinetics of tigecycline are unclear, and the optimal dosing strategy is controversial.

**Methods:** A single-center prospective clinical study that included critically ill patients who received tigecycline was performed. Blood samples were intensively sampled (eight samples each), and plasma drug concentrations were determined. A population pharmacokinetic (PPK) model was developed and evaluated by goodness-of-fit plots, bootstrap analysis and visual predictive checks. Monte Carlo simulation was conducted to optimize the dosage regimen.

**Results:** Overall, 751 observations from 98 patients were included. The final PPK model was a two-compartment model incorporating covariates of creatinine clearance on clearance (CL), body weight on both central and peripheral volumes of distribution (V1 and V2), γ-glutamyl transferase and total bilirubin on intercompartment clearance (Q), and albumin on V2. The typical values of CL, Q, V1 and V2 were 3.09 L/h, 39.7 L/h, 32.1 L and 113 L, respectively. A dosage regimen of 50 mg/12 h was suitable for complicated intra-abdominal infections, but 100 mg/12 h was needed for community-acquired pneumonia, skin and skin structure infections and infections caused by less-susceptive bacteria.

**Conclusion:** The Tigecycline PPK model was successfully developed and validated. Individualized dosing of tigecycline could be beneficial for critically ill patients.

## Introduction

The increasing incidence of drug-resistant bacterial infections has become one of the major threats to human health (Hu et al., 2018; World Health Organization, 2022). In China alone, 27.45% of bacterial infections in inpatients were drug resistant, 15.77% of which were multidrug resistant (Zhen et al., 2021). The prevalence in critically ill patients was even higher. In developed countries, approximately 25% of critically ill patients experience at least one healthcare-associated infection, while the rate is as high as 50% in developing countries (Vincent et al., 2020; Wang et al., 2022). In addition, a global estimation indicated that approximately 4.95 million deaths in 2019 were associated with antimicrobial resistance, and this number is projected to increase to more than 10 million by 2050 (Murray et al., 2022). Tigecycline, as the first glycylcycline antimicrobial drug approved by the FDA in 2005, has broad-spectrum antimicrobial activity, including multidrug-resistant Gram-negative bacteria (Peterson, 2008; Yaghoubi et al., 2022). Thus, tigecycline alone or a tigecycline-based combination regimen are often considered one of the last resorts when other alternative antibiotics are not suitable for infections caused by multidrug-resistant bacteria (Peterson, 2008; Tamma et al., 2023).

The study of antimicrobial pharmacokinetics/pharmacodynamics (PK/PD) involves the combination of drug concentration, time, and antibacterial activity, which characterize the time course of antimicrobial effects in the body (Roberts et al., 2015). This approach provides a promising new approach to drug dosage adjustment in patients with severe infections, which is particularly appropriate for analyzing and predicting changes in drugs with large individual differences and is one of the reliable strategies for realizing the therapeutic potential of preexisting antimicrobial drugs that are in clinical use (Kalil et al., 2016). However, the PK/PD evidence for tigecycline in critically ill patients is insufficient. Most of the published population pharmacokinetic (PPK) studies on tigecycline were conducted in special subpopulations (e.g., patients with intra-abdominal infections, hospital-acquired pneumonia, sepsis or septic shock, as well as patients with decompensated cirrhosis and severe infections, etc.), while only two studies with limited sampling were carried out on critically ill patients (Wart et al., 2006; Rubino et al., 2010; Xie et al., 2017; Broeker et al., 2018; Moor et al., 2018; Yang et al., 2021; Zhou et al., 2021; Amann et al., 2022; Bastida et al., 2022; Luo et al., 2023). On the other hand, although the recommended dose of tigecycline in drug labels appears to be straightforward (an initial dose of 100 mg followed by 50 mg every 12 h for 5–14 days), increasing evidence shows that the common dose may result in higher all-cause mortality, whereas a high-dose tigecycline therapy strategy (an initial dose of 200 mg followed by 100 mg every 12 h) may have higher clinical cure rate (McGovern et al., 2013; Xie et al., 2014; Zha et al., 2020). Therefore, there is controversy about the optimal dosing strategy for tigecycline. Tigecycline has time-dependent antimicrobial activity with a long postantibiotic effect, and its clinical and microbiological efficacy has been related to the area under the curve/minimum inhibitory concentration (AUC/MIC), while the target value varies according to the infections (Meagher et al., 2007; Passarell et al., 2008; Xie et al., 2017; Leng et al., 2021). Moreover, the individual variations in the pharmacokinetic parameters of tigecycline observed were very large (the typical values of clearance and central volumes of distribution of tigecycline ranged from 7.5 to 23.1 L/h and 58.7–162 L, respectively), which was further exacerbated by the special pathophysiology of critically ill patients, such as liver dysfunction, hemodynamic changes, and hypoproteinemia (Wart et al., 2007; Zhou et al., 2022).

Hence, in this prospective study, with an intensive sampling strategy, we tried to develop a PPK model of tigecycline in critically ill patients, identify the patient factors influencing pharmacokinetics, and utilize the resultant model to describe optimized tigecycline dosage regimens for different bacterial infections.

## Methods

### Study design and ethics approval

This single-center prospective study was conducted in accordance with the Declaration of Helsinki and approved by the ethics committee of Sir Run Run Shaw Hospital, School of Medicine, Zhejiang University (reference number KEYAN20190108-9). Informed consent was obtained from the patient or his/her representative before enrollment.

### Patient inclusion, drug administration and sample collection

We included critically ill patients who received intermittent intravenous tigecycline therapy between July 2019 and July 2023. The inclusion and exclusion criteria for inpatients are shown in Supplementary Table S2. Tigecycline was administered every 12 h intermittently intravenously as a 30-min infusion. The dose of tigecycline was decided upon by the physician based on clinical evaluation. Blood samples for pharmacokinetic analysis were collected immediately before the seventh drug administration and at 0.5, 1, 2, 3, 4, 6, and 12 h postdose. The plasma samples were obtained after centrifugation and analyzed via a validated LC‒MS/MS method (Shao et al., 2017).

We also recorded the following data items for model development: age, sex, body weight (BW), height and body mass index (BMI), infection site, infection pathogen, tigecycline dosing regimen, serum creatinine (SCr), baseline albumin (ALB), blood urea nitrogen (BUN), alanine aminotransferase (ALT), aspartate aminotransferase (AST), total bilirubin (TBIL), alkaline phosphatase (ALP), γ-glutamyl transferase (GGT), white blood cell (WBC), red blood cell (RBC) and platelet levels. The creatinine clearance (CCr) was calculated according to the Cockcroft-Gault equation (Cockcroft and Gault, 1976).

### PK analyses and PPK modeling

PK analysis was performed using the nonlinear mixed-effects modeling programs NONMEM (v7.5.0, ICON, Ellicott City, MD, United States) and PDxPop (v5.3.1, ICON, Gaithersburg, MD, United States). The graphical visualizations were performed with the R program (v4.2.3; https://www.r-project.org/).

Models were developed and evaluated based on the objective function value (OFV), Akaike information criterion (AIC), and goodness-of-fit plots. In reference to previous studies, the one- and two-compartment models were tested as the base model (Zhou et al., 2022). The interindividual variability was modeled exponentially, while the residual variability with additive, proportional, and combined error models was evaluated with the first-order conditional estimation with interaction method (FOCE-I). Forward inclusion and backward elimination methods were used to confirm the significant covariates. Continuous covariates, such as patient age, BW, BMI, renal function (CCr, BUN), and liver function (ALB, AST, ALT, ALP, GGT, TBIL), were tested with linear (Eq. 1), exponential (Eq. 2) and power models (Eqs. 3, 4).

$$\theta_{i}=\theta_{pop}+\theta_{cov}\times \left(\frac{{cov}_{i}}{{cov}_{median}}\right)$$ (1)

$$\theta_{i}=\theta_{pop}\times e^{\theta_{cov}\times \left(\frac{{cov}_{i}}{{cov}_{median}}\right)}$$ (2)

$$\theta_{i}=\theta_{pop}\times {\left(\frac{{cov}_{i}}{{cov}_{median}}\right)}^{\theta_{cov}}$$ (3)

$$\theta_{i}=\theta_{pop}\times {\theta_{cov}}^{\left(\frac{{cov}_{i}}{{cov}_{median}}\right)}$$ (4)

where θ_i_ is the individual parameter for patient i, θ_pop_ is the typical value of the pharmacokinetic parameter, cov_i_ is the covariate value normalized by the population median value (cov_median_), and θcov is the covariate effect.

The categorical covariates (e.g., sex, daily dose of tigecycline) were modeled using a power model as follows (Eq. 5):

$$\theta_{i}=\theta_{pop}\times {\left(\theta_{cov}\right)}^{{cov}_{i}}$$ (5)

where cov_i_ is the categorical covariate value (0 or 1) for individual patient i, and θ_pop_ is the typical value of the pharmacokinetic parameter when cov_i_ = 0.

Correlation analysis was performed before covariate model development to ensure that the final model did not contain correlated or covariate variables. Covariates were included in the model based on the criteria of OFV requiring a decrease of 3.84 (*p* < 0.05) in forward inclusion and an increase of greater than 10.83 (*p* < 0.001) in backward elimination. Goodness-of-fit plots, bootstrap analysis, and visual predictive checks (VPCs) were utilized to evaluate the final model and parameter estimates. For bootstrap analysis, the resampling process was repeated 1,000 times to assess the robustness and reliability of the final model. Subsequently, the PK parameter estimates derived from these resampled data sets were compared with the original PK estimates from the final model utilizing the median and 95% confidence intervals (Ette and Onyiah, 2002; Ette et al., 2003). For VPCs, 1,000 simulated replicates of the original dataset were performed to evaluate the predictive performance of the final model. The median, fifth, and 95th percentiles of the simulated concentrations were calculated, and the 95% prediction interval for each bin was also derived (Bergstrand et al., 2011).

### Simulation and dosing regimen optimization

Monte Carlo simulation (MCS) was performed for the probability of target attainment (PTA) analysis based on the final model. Two sets of dose simulations were performed: 50 mg/12 h for the conventional dose group and 100 mg/12 h for the high-dose group. Based on the covariates that were included in the final model, the simulations were stratified by CCr group (30, 80, and 130 mL/min), BW group (40, 60, 80, and 100 kg) and liver function group (normal and abnormal). The MIC was set at 0.125, 0.25, 0.5, 1, 2, 4, and 8 mg/L. Each group consisted of 1,000 virtual patients. The AUC was calculated using the pkr package (version 0.1.3) in the R program with linear-up and linear-down methods. Previous studies have used a classification and regression tree approach (CART) to identify the different PK/PD targets to improve the microbiological and clinical responses to different types of infections, which were evaluated in this study (Meagher et al., 2007; Passarell et al., 2008; Xie et al., 2017; Leng et al., 2021): (i) AUC/MIC≥6.96 for complicated intra-abdominal infection (cIAI); (ii) AUC/MIC≥12.8 for community-acquired bacterial pneumonia (CAP); and (iii) AUC/MIC≥17.9 for complicated skin and skin structure infection (cSSSI). Optimal dosing regimens were chosen according to their capacity to attain a PTA of 90%.

## Results

### Patient inclusion and characteristics

In total, 98 patients were enrolled, and 751 tigecycline concentration levels were obtained to develop the PPK model. The demographic characteristics of the included patients are shown in Table 1.

**TABLE 1:** Demographic characteristics of the included patients.

Variable	Total (*n* = 98)	Min–Max
Sex		
Male	65 (66.3%)	
Female	33 (33.7%)	
Age (years)	63.4 ± 18.0	20–92
Weight (kg)	62.3 ± 12.4	38–92.5
BMI (kg/m^2^)	22.7 ± 3.72	13.9–31.1
Infected site, n (%)		
Intra-abdominal infection	37 (37.8%)	
Pulmonary infection	31 (31.6%)	
Skin and soft tissue infection	6 (6.12%)	
Bloodstream infection	9 (9.18%)	
Trauma	7 (7.14%)	
Others	22 (22.4%)	
Pathogen		
CRAB	46 (46.9%)	
CRKP	24 (24.5%)	
CRPA	2 (2.04%)	
CRE	1 (1.02%)	
MRSA	1 (1.02%)	
ESBL+	2 (2.04%)	
Other	25 (25.5%)	
undefined	7 (7.14%)	
MIC		
0.12 mg/L	5 (5.10%)	
0.25 mg/L	1 (1.02%)	
0.5 mg/L	22 (22.4%)	
1 mg/L	38 (38.8%)	
2 mg/L	19 (19.4%)	
4 mg/L	2 (2.04%)	
undefined	11 (11.2%)	
Daily dose		
100 mg/d	91 (92.9%)	
200 mg/d	7 (7.14%)	
Treatment duration (day)	12 (8,20)	4–68
CCr (mL/min)	77.0 (51.1,142)	6.40–338
Laboratory data		
BUN (mmol/L)	10.8 (7.29,17.0)	1.46–44.8
SCr (μmol/L)	64.0 (44.0,103)	17.0–565
TP (g/L)	51.1 (46.2,56.2)	7.40–81.2
ALB (g/L)	27.9 (25.8,30.3)	17.3–38.3
ALT (U/L)	19.0 (12.0,34.0)	2.00–1974
AST (U/L)	24.0 (18.0,37.0)	7.00–829
ALP (U/L)	99.0 (75.0,139)	31.0–2548
GGT (U/L)	53.0 (30.0,103)	8.00–1088
TBIL (μmol/L)	15.7 (10.0,30.4)	4.00–144
PT (s)	16.5 (15.1,17.9)	12.6–30.5
WBC (10^9^/L)	8.30 (5.10,12.0)	0.100–43.4
RBC (10^12^/L)	2.53 (2.32,2.88)	1.62–4.52
PLT (10^9^/L)	139 (68.0,225)	1.00–615

All covariates data were measured on the day of concentration monitoring. All continuous data are presented as the mean ± standard deviation or median and interquartile range, and categorical data are presented as numbers and percentages.

Abbreviations: BMI, body mass index; CRAB, carbapenem-resistant *Acinetobacter baumannii*; CRKP, carbapenem-resistant *Klebsiella pneumoniae*; CRPA, carbapenem-resistant *Pseudomonas aeruginosa*; CRE, carbapenem-resistant Enterobacteriaceae; MRSA, methicillin-resistant *Staphylococcus aureus*; ESBL, extended spectrum beta-lactamase; MIC, minimum inhibitory concentration; ECMO, extracorporeal membrane oxygenation; CCr, creatinine clearance; BUN, blood urea nitrogen; SCr, serum creatinine; TP, total protein; ALB, albumin; ALT, alanine aminotransferase; AST, aspartate aminotransferase; ALP, alkaline phosphatase; GGT, γ-glutamyl transpeptidase; TBIL, total bilirubin; PT, prothrombin time; WBC, white blood cell; RBC, red blood cell; PLT, platelet.

### PPK model development

**FIGURE 1:**
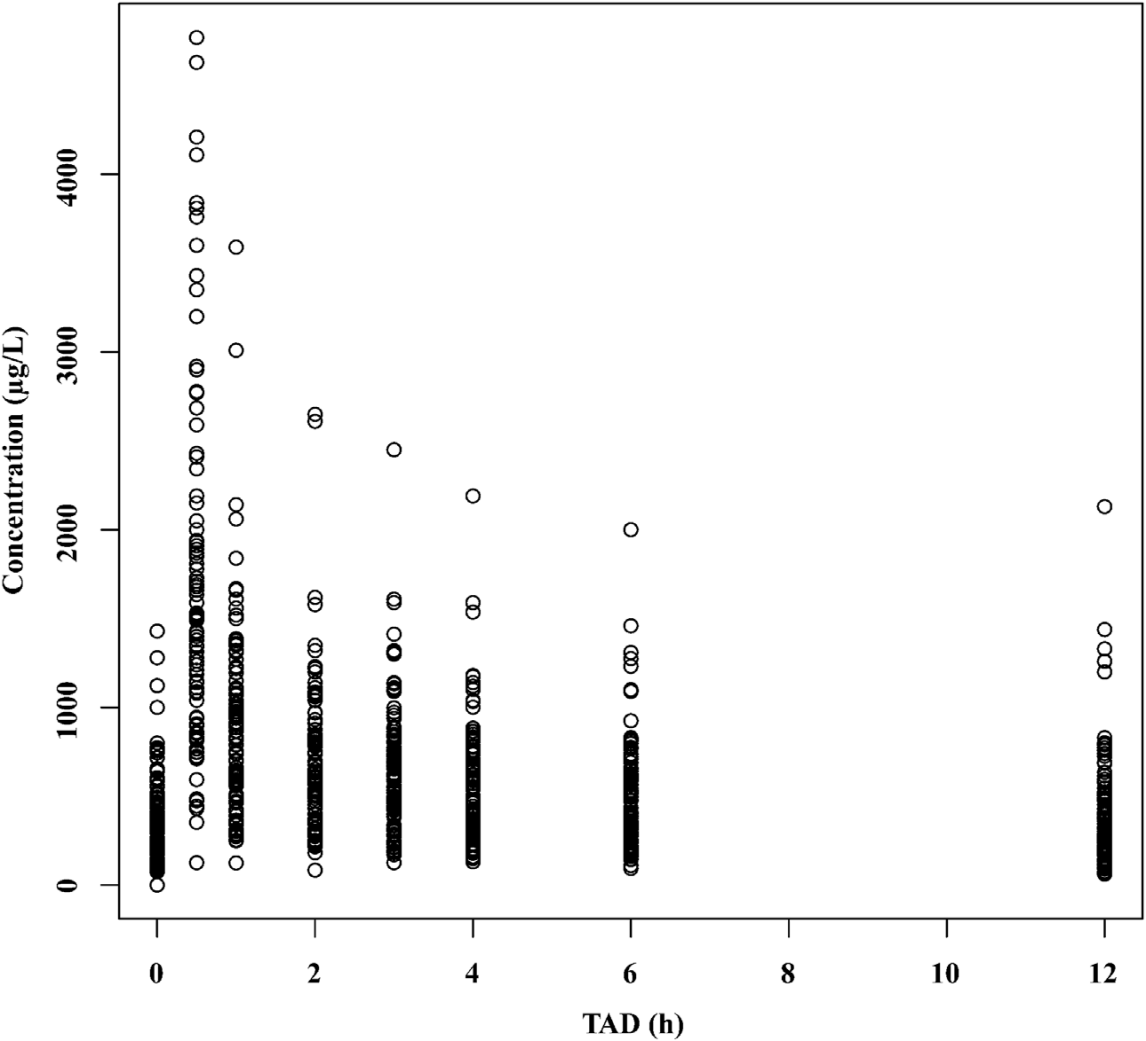
The concentration–time profile of tigecycline. TAD, time after dose.

The concentration-time profile of tigecycline is shown in Figure 1. The tigecycline concentration data could be well illustrated by a two-compartment model with first-order elimination (9325.681 and 8242.914 for AIC in one- and two-compartments, respectively). An exponential model and a proportional model were used to describe the interindividual variability and residual variability, respectively. The final PPK model and parameter estimates are shown in Table 2, and the key progression of covariate screening is shown in Supplementary Table S3. The inclusion of CCr in the clearance (CL) test in the final PPK model decreased the OFV by 38.968. The other four covariates [GGT and TBIL on intercompartment clearance (Q), ALB on peripheral volumes of distribution (V2), and BW on both central and peripheral volumes of distribution (V1 and V2)] were also found to be significant. However, it was not possible to estimate the interindividual variability for V2. The typical values of CL, Q, V1 and V2 were 3.09 L/h, 39.7 L/h, 32.1 L and 113 L, respectively.

**TABLE 2:** Distribution characteristics of the final model estimation parameters and bootstrap analysis.

Parameter	Final model	Bootstrap analysis	
	Estimate [RSE (%)]	Median estimate [RSE (%)]	95%CI
CL (L/h)	3.09 (26.3)	3.10 (18.6)	1.98–4.24
V1 (L)	32.1 (8.38)	31.0 (10.1)	25.8–37.9
Q (L/h)	39.7 (5.79)	40.0 (6.41)	35.0–45.5
V2 (L)	113 (4.61)	110 (5.18)	101–124
θ_CLCR-CL_	3.28 (27.9)	3.31 (17.8)	1.01–2.92
θ_BW-V1_	1.95 (28.8)	1.96 (24.3)	1.15–2.78
θ_GGT-Q_	0.956 (32.8)	0.987 (34.1)	0.240–1.57
θ_TBIL-Q_	−0.912 (33.3)	−0.900 (32.7)	−1.47–−0.304
θ_BW-V2_	1.61 (13.1)	1.60 (14.3)	1.13–2.06
θ_ALB-V2_	4.52 (23.0)	4.33 (25.7)	1.78–6.23
Interindividual variability			
ω_CL_ (%)	27.0 (15.0)	25.8 (15.5)	19.2–34.6
ω_V1_ (%)	72.5 (13.9)	70.9 (13.3)	53.1–89.5
ω_Q_ (%)	18.8 (39.5)	16.4 (44.2)	4.42–31.2
Residual variability			
σ (%)	2.02 (13.8)	2.01 (12.8)	1.57–2.60

The final model:

CL (L/h) = 3.09+(CCr/77)×3.28,

V1 (L) = 32.1×(BW/61)^1.95^,

Q (L/h) = 39.7×(logGGT/1.7)^0.956^×(logTBIL/1.2)^−0.912^,

V2 (L) = 113×(BW/61)^1.61^×(logALB/1.4)^4.52^.

Abbreviations: CL, typical value of apparent clearance; V1, central volume of distribution; Q, intercompartmental clearance; V2, peripheral volume of distribution; θ, fixed-effect parameter; CCr, creatinine clearance; BW, body weight; σ, residual variability for proportional error; RSE, residual standard error; CI, confidence interval.

### Model evaluation

**FIGURE 2:**
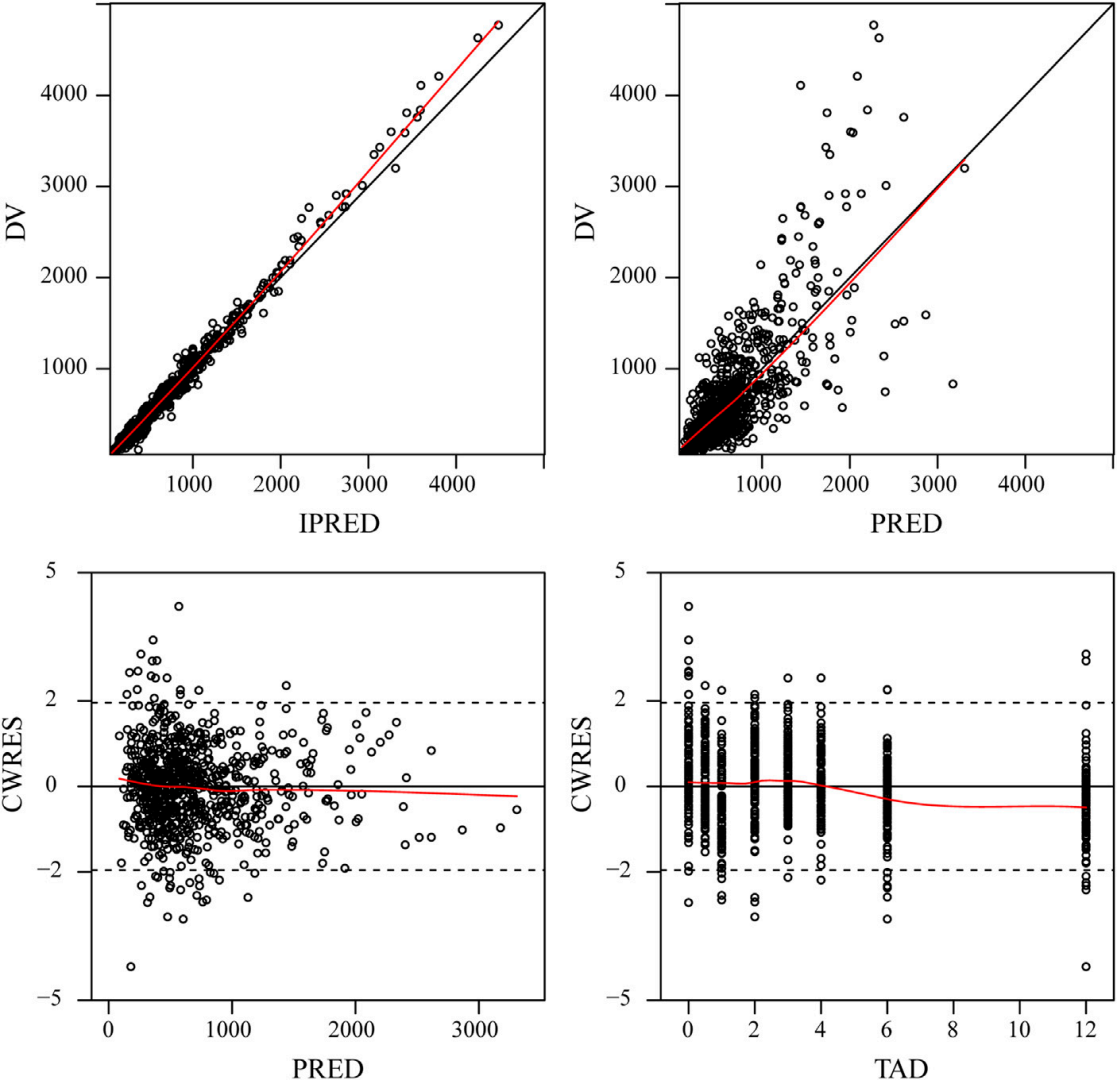
Goodness-of-fit plots for the final PPK model. Observed tigecycline concentrations (DV) *versus* individual predictions (IPRED); DV *versus* population predictions (PRED); Conditional weighted residuals (CWRES) *versus* PRED; CWRES *versus* time after dose (TAD).

**FIGURE 3:**
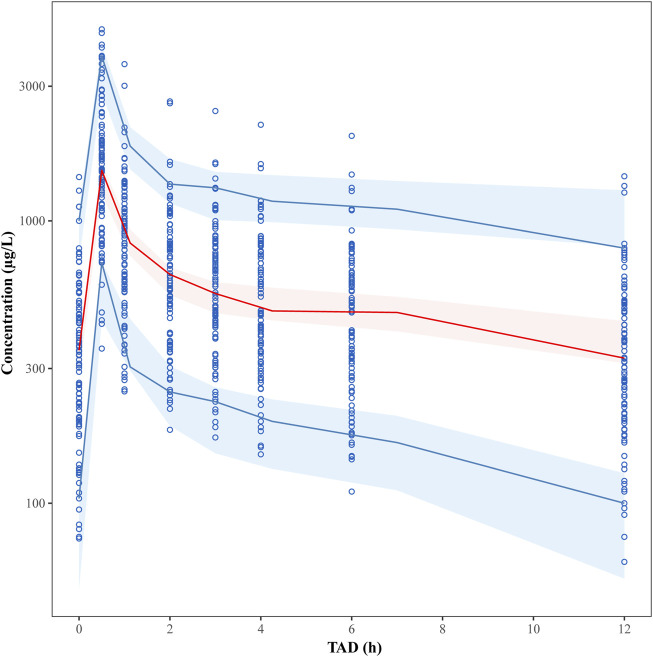
Visual predictive check (VPC) plots for the plasma tigecycline concentration. The *Y*-axis is the logarithm of the concentration. The open circles represent the observed concentrations. The solid red line represents the median of the observations. The solid blue lines represent the 5th and 95th percentiles of the observations. The blue shaded areas represent the 95% confidence intervals for the 5th and 95th percentiles, and the red shaded areas represent the medians of the predicted data.

The final model was evaluated by goodness-of-fit plots (Figure 2) and VPCs (Figure 3), which showed that the model had good predictive performance. The 1,000 bootstrap analyses are presented in Table 2, which indicates the robustness of the final model.

### Simulation and dosing regimen optimization

**FIGURE 4:**
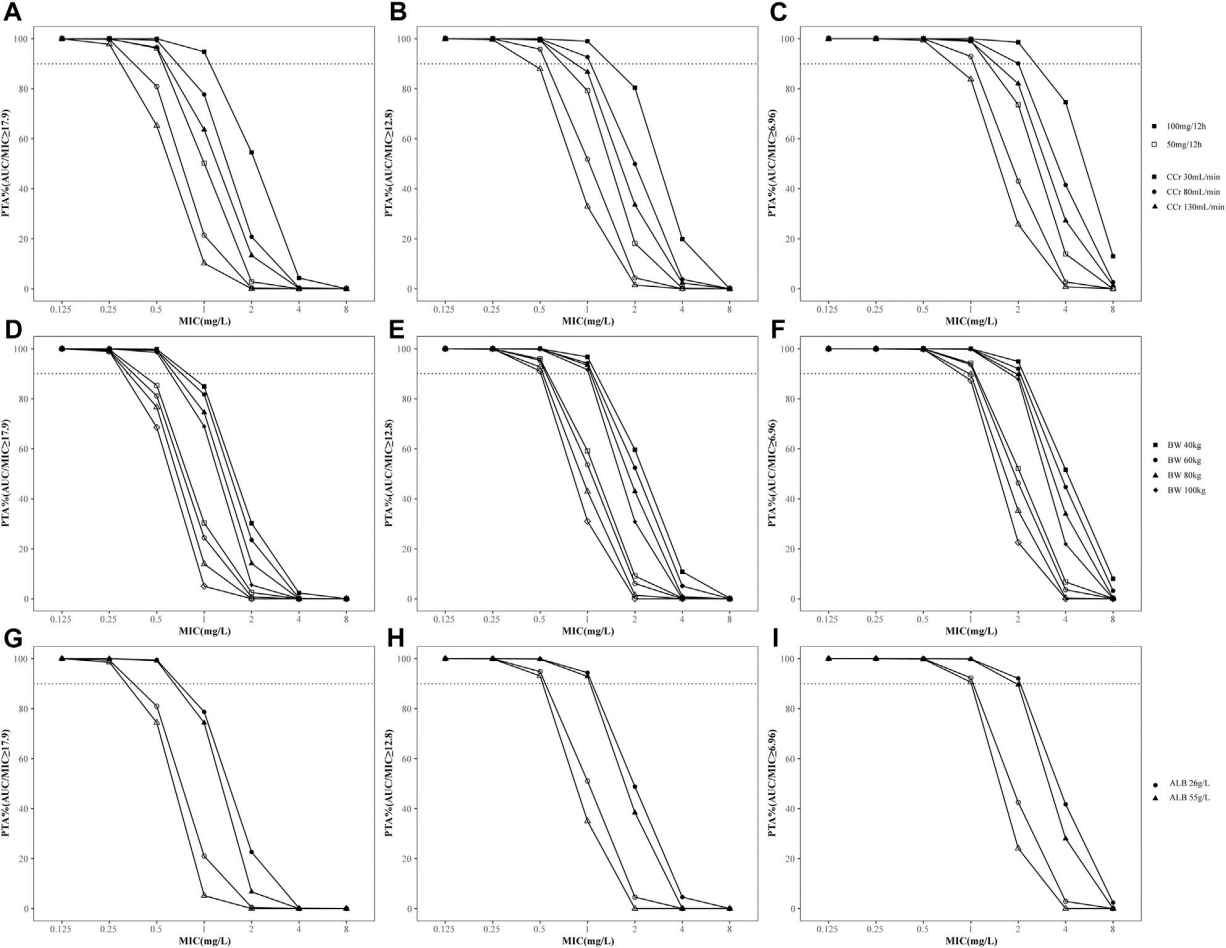
Probability of target attainment (PTA) for different dosage regimens of tigecycline for PK/PD targets with AUC/MIC≥6.96, 12.8 and 17.9. **(A–C)** PTA stratified by creatinine clearance; **(D–F)** PTA stratified by body weight; **(G–I)** PTA stratified by albumin concentration. The dashed lines indicate a PTA of 90%. The solid icons represent the high-dose regimen (100 mg every 12 h). Hollow icons represent the standard dosage regimen (50 mg every 12 h).

The PTAs for different dosages of tigecycline for critically ill patients according to the MCS are shown in Figure 4. The PTA decreased when CCr, BW or ALB increased. Based on the simulation results, the standard dosage regimen was sufficient to achieve an AUC/MIC ratio of 6.96 at MICs ≤1 mg/L, while the desired PTA may not be achieved even with higher doses in patients with augmented renal clearance (CCr >130 mL/min). In contrast, for therapeutic AUC/MIC values of 12.8 and 17.9, the standard dosage regimen may attain the suboptimal target at MICs >0.5 mg/L or 0.25 mg/L, respectively. Therefore, a dosage regimen of 50 mg every 12 h was suitable for complicated intra-abdominal infections, but 100 mg every 12 h was needed for community-acquired pneumonia, skin and skin structure infections and infections caused by less-susceptible bacteria, which was defined as intermediate to tigecycline. We also compared the PTAs in patients with normal or abnormal TBIL or GGT levels [TBIL>2 × upper limit of normal (ULN) or GGT>3×ULN] to examine the effect of liver function on tigecycline efficacy (Andrade et al., 2019; Lu and Chinese Society of Hepatology and Chinese Medical Association, 2022). The results showed that the PTA was not altered by the increase in TBIL or GGT (Supplementary Figure S3).

## Discussion

To our knowledge, this was the largest single-center prospective clinical study using an intensive sampling strategy to date to develop the tigecycline PPK model in critically ill adult patients. Due to the large sample size and uniformly distributed sampling times during the dosing interval, compartment model selection and covariate inclusion would be informative for individualized dosing and future studies. Finally, a two-compartment PPK model with up to 5 covariates was developed and validated.

The estimated values of CL (3.09 L/h) and V1 (32.1 L) of tigecycline were lower than those estimated in previously published studies; however, the estimated values of Q (39.7 L/h) and V2 (113 L) were similar to those of other studies. The large variations in CL and V1 observed in the included populations were most likely due to intra- and interindividual variations in subjects with different physical-pathological statuses. This finding illustrates the importance of performing individualized dosing.

In this study, we found that CCr and BW were the two significant covariates of CL and V, respectively, in the final tigecycline PPK model. Approximately 22% of tigecycline is excreted by the kidneys, so a patient’s creatinine clearance can significantly impact tigecycline elimination (Peterson, 2008). Two previous studies ultimately included CCr as a covariate, and CCr was positively correlated with the clearance of tigecycline (Wart et al., 2006; Rubino et al., 2010). Another study of the population pharmacokinetics of tigecycline in hospital-acquired pneumonia patients identified SCr as a significant predictor of CL in the final PPK model, although they did not investigate the possible effect of CCr (Zhou et al., 2021). The effect of BW as a covariate on the pharmacokinetics of tigecycline has also been studied previously, and the results showed that BW may affect both the clearance and apparent volume of distribution of tigecycline (Zhou et al., 2021). The simulation results indicated that the tigecycline dose did not need to be adjusted based on CCr or BW from the perspective of PTA, which was consistent with the current recommendation. However, caution should be taken when treating patients with augmented renal clearance (CCr >130 mL/min) and obesity, as tigecycline exposure is less common than needed.

On the other hand, tigecycline is metabolized by the liver, so the clearance of tigecycline is theoretically related to the patient’s liver function (Amann et al., 2022). Although the label of tigecycline has already been suggested to half the maintenance dose for patients with liver cirrhosis with a Child-Pugh score of C, it remains unknown whether the dose should be adjusted for patients with impaired liver function but without cirrhosis, as these conditions are common in clinical practice. We included liver function indicators (TBIL, GGT, and ALB) as covariates in the final model. However, the simulation results showed that elevated TBIL and GGT levels did not influence the PTA of tigecycline, which suggested that the inclusion of these covariates may not be clinically significant. Therefore, tigecycline may not need dose adjustment in patients with liver function impairment in clinical practice. This result is also supported. Most studies have screened liver function indicators as potential covariates, but only four studies included the effect of TBIL, bilirubin, BUN or AST on clearance in the final PPK model, and none of the studies showed a significant effect on intercompartment clearance (Broeker et al., 2018; Yang et al., 2021; Zhou et al., 2021; Amann et al., 2022). In addition, the final model showed a significant effect of ALB on V2, which may be related to its high protein binding of tigecycline, although no studies included ALB in the final model. Another pharmacokinetic study of critically ill patients with decompensated cirrhosis and severe infections revealed that patients with hypoproteinaemia have similar steady-state concentrations compared with patients with normoproteinaemia, although the total serum protein concentration was found to be correlated with V1 (Bastida et al., 2022). Therefore, some researchers believe that the effects of liver function parameters on tigecycline pharmacokinetics are likely minimal.

In addition, Monte Carlo simulations were performed based on different doses and MICs for different infections. The results showed that high-dose tigecycline significantly increased PTA in patients with various infections, especially in patients with drug-resistant bacterial infections; this finding was intuitive and was proposed for many other antibiotics. Some studies have also shown that the conventional dose (50 mg every 12 h) of tigecycline was often insufficient for reaching therapeutic target, especially in critically ill patients with multidrug-resistant bacterial infections, which might be the main reason for the increase in the all-cause mortality rate in patients receiving tigecycline treatment for severe infections and sepsis (Pascale et al., 2014; US, F. and D. A., 2010). Furthermore, a systemic review revealed that high-dose (100 mg every 12 h) tigecycline therapy may lower all-cause mortality and increase clinical cure and microbial clearance rates in critically ill patients without an increased incidence of adverse effects compared to conventional dosage regimens (Zha et al., 2020). Our results support the use of high-dose tigecycline, except for in patients with intra-abdominal infection. The PTA of conventional dosing for cIAI was satisfactory, possibly due to the high distribution of tigecycline in abdominal tissues and the low PK/PD target of this type of infection. Due to the liver toxicity of tigecycline, individualized dosing considering the severity of infection, infection status, and infection site is needed (Yu et al., 2022).

This study has several limitations. Tigecycline exhibits atypical nonlinear plasma protein binding (PPB) behavior, and PK/PD target attainment analysis for the determination of clinical breakpoints focused on the total AUC without accounting for the PPB of tigecycline should be evaluated in future clinical studies to determine the *f*AUC/MIC. Although a higher dose of tigecycline has been recommended for some populations, studies assessing the efficacy and safety of high-dose tigecycline in severe infections are still limited and remain controversial, and this model lacks a large number of patients for external validation; furthermore, for conclusive evidence on the optimal dosing strategy, well-designed randomized studies are needed.

## Conclusion

This was a single-center prospective study in which a tigecycline PPK model was developed for critically ill patients using an intensive sampling strategy. The pharmacokinetics of tigecycline in this population were well described by a two-compartment model with the significant covariates CCr on CL, body weight on both V1 and V2, GGT and TBIL on Q, and ALB on V2. A dose recommendation was also made based on the model simulation, which suggested that a dosage regimen of 50 mg every 12 h was suitable for complicated intra-abdominal infections, and 100 mg every 12 h was needed for community-acquired pneumonia, skin and skin structure infections and infections caused by less-susceptive bacteria. This provided important evidence for tigecycline dosage individualization in these populations.

## Data Availability

The original contributions presented in the study are included in the article/Supplementary Material, further inquiries can be directed to the corresponding authors.

## References

[B1] Amann L. F. Alraish R. Broeker A. Kaffarnik M. Wicha S. G. (2022). Tigecycline dosing strategies in critically ill liver-impaired patients. Antibiotics 11, 479. 10.3390/antibiotics11040479 35453230 PMC9028393

[B2] Andrade R. J. Aithal G. P. Björnsson E. S. Kaplowitz N. Kullak-Ublick G. A. Larrey D. (2019). EASL clinical practice guidelines: drug-induced liver injury. J. Hepatology 70, 1222–1261. 10.1016/j.jhep.2019.02.014 30926241

[B3] Bastida C. Hernández-Tejero M. Cariqueo M. Aziz F. Fortuna V. Sanz M. (2022). Tigecycline population pharmacokinetics in critically ill patients with decompensated cirrhosis and severe infections. J. Antimicrob. Chemother. 77, 1365–1371. 10.1093/jac/dkac036 35178567

[B4] Bergstrand M. Hooker A. C. Wallin J. E. Karlsson M. O. (2011). Prediction-corrected visual predictive checks for diagnosing nonlinear mixed-effects models. AAPS J. 13, 143–151. 10.1208/s12248-011-9255-z 21302010 PMC3085712

[B5] Broeker A. Wicha S. G. Dorn C. Kratzer A. Schleibinger M. Kees F. (2018). Tigecycline in critically ill patients on continuous renal replacement therapy: a population pharmacokinetic study. Crit. Care 22, 341. 10.1186/s13054-018-2278-4 30558639 PMC6296114

[B6] Cockcroft D. W. Gault H. (1976). Prediction of creatinine clearance from serum creatinine. Nephron 16, 31–41. 10.1159/000180580 1244564

[B7] Ette E. I. Onyiah L. C. (2002). Estimating inestimable standard errors in population pharmacokinetic studies: the bootstrap with winsorization. Eur. J. Drug Metab. Pharmacokinet. 27, 213–224. 10.1007/BF03190460 12365204

[B8] Ette E. I. Williams P. J. Kim Y. H. Lane J. R. Liu M. Capparelli E. V. (2003). Model appropriateness and population pharmacokinetic modeling. J. Clin. Pharma 43, 610–623. 10.1177/0091270003253624 12817524

[B9] Hu F. Zhu D. Wang F. Wang M. (2018). Current status and trends of antibacterial resistance in China. Clin. Infect. Dis. 67, S128–S134. 10.1093/cid/ciy657 30423045

[B10] Kalil A. C. Metersky M. L. Klompas M. Muscedere J. Sweeney D. A. Palmer L. B. (2016). Management of adults with hospital-acquired and ventilator-associated pneumonia: 2016 clinical practice guidelines by the infectious diseases society of America and the American thoracic society. Clin. Infect. Dis. 63, e61–e111. 10.1093/cid/ciw353 27418577 PMC4981759

[B11] Leng B. Yan G. Wang C. Shen C. Zhang W. Wang W. (2021). Dose optimisation based on pharmacokinetic/pharmacodynamic target of tigecycline. J. Glob. Antimicrob. Resist. 25, 315–322. 10.1016/j.jgar.2021.04.006 33957288

[B12] Lu L. Chinese Society of Hepatology and Chinese Medical Association (2022). Guidelines for the management of cholestatic liver diseases (2021). J. Clin. Transl. Hepatol. 10, 757–769. 10.14218/JCTH.2022.00147 36062287 PMC9396310

[B13] Luo X. Wang S. Li D. Wen J. Sun N. Fan G. (2023). Population pharmacokinetics of tigecycline in critically ill patients. Front. Pharmacol. 14, 1083464. 10.3389/fphar.2023.1083464 36992827 PMC10040605

[B14] McGovern P. C. Wible M. El-Tahtawy A. Biswas P. Meyer R. D. (2013). All-cause mortality imbalance in the tigecycline phase 3 and 4 clinical trials. Int. J. Antimicrob. Agents 41, 463–467. 10.1016/j.ijantimicag.2013.01.020 23537581

[B15] Meagher A. K. Passarell J. A. Cirincione B. B. Wart S. A. V. Liolios K. Babinchak T. (2007). Exposure-response analyses of tigecycline efficacy in patients with complicated skin and skin-structure infections. Antimicrob. Agents Chemother. 51, 1939–1945. 10.1128/AAC.01084-06 17353238 PMC1891381

[B16] Moor A. B.-D. Rypulak E. Potręć B. Piwowarczyk P. Borys M. Sysiak J. (2018). Population pharmacokinetics of high-dose tigecycline in patients with sepsis or septic shock. Antimicrob. Agents Chemother. 62, e02273-17. 10.1128/aac.02273-17 29358291 PMC5913959

[B17] Murray C. J. L. Ikuta K. S. Sharara F. Swetschinski L. Robles Aguilar G. Gray A. (2022). Global burden of bacterial antimicrobial resistance in 2019: a systematic analysis. Lancet 399, 629–655. 10.1016/S0140-6736(21)02724-0 35065702 PMC8841637

[B18] Pascale G. D. Montini L. Pennisi M. Bernini V. Maviglia R. Bello G. (2014). High dose tigecycline in critically ill patients with severe infections due to multidrug-resistant bacteria. Crit. Care 18, 1–9. 10.1186/cc13858 PMC4057423 24887101

[B19] Passarell J. A. Meagher A. K. Liolios K. Cirincione B. B. Wart S. A. V. Babinchak T. (2008). Exposure-response analyses of tigecycline efficacy in patients with complicated intra-abdominal infections. Antimicrob. Agents Chemother. 52, 204–210. 10.1128/AAC.00813-07 17954694 PMC2223921

[B20] Peterson L. R. (2008). A review of tigecycline — the first glycylcycline. Int. J. Antimicrob. Agents 32, S215–S222. 10.1016/S0924-8579(09)70005-6 19134522

[B21] Roberts J. A. Taccone F. S. Lipman J. (2015). Understanding PK/PD. Intensive Care Med. 42, 1797–1800. 10.1007/s00134-015-4032-6 26334756

[B22] Rubino C. M. Forrest A. Bhavnani S. M. Dukart G. Cooper A. Korth-Bradley J. (2010). Tigecycline population pharmacokinetics in patients with community- or hospital-acquired pneumonia. Antimicrob. Agents Chemother. 54, 5180–5186. 10.1128/AAC.01414-09 20921315 PMC2981274

[B23] Shao R. Li X. Hu Y. Chen J. Lou H. Dai H. (2017). Determination of tigecycline in human plasma by LC-MS/MS and its application to population pharmacokinetics study in Chinese patients with hospital-acquired pneumonia. Biomed. Chromatogr. 32, e4045. 10.1002/bmc.4045 28677837

[B24] Tamma P. D. Aitken S. L. Bonomo R. A. Mathers A. J. Duin D. van Clancy C. J. (2023). Infectious diseases society of America 2023 guidance on the treatment of antimicrobial resistant gram-negative infections. Clin. Infect. Dis., ciad428. 10.1093/cid/ciad428 37463564

[B25] US, F. and D. A. (2010). FDA drug safety communication: increased risk of death with Tygacil (tigecycline) compared to other antibiotics used to treat similar Infections.

[B26] Vincent J.-L. Sakr Y. Singer M. Martin-Loeches I. Machado F. R. Marshall J. C. (2020). Prevalence and outcomes of infection among patients in intensive Care units in 2017. JAMA 323, 1478–1487. 10.1001/jama.2020.2717 32207816 PMC7093816

[B27] Wang Y. Xiao Y. Yang Q. Wang F. Wang Y. Yuan C. (2022). Clinical prediction models for multidrug-resistant organism colonisation or infection in critically ill patients: a systematic review protocol. BMJ Open 12, e064566. 10.1136/bmjopen-2022-064566 PMC9528596 36175101

[B28] Wart S. A. V. Cirincione B. B. Ludwig E. A. Meagher A. K. Korth-Bradley J. M. Owen J. S. (2007). Population pharmacokinetics of tigecycline in healthy volunteers. J. Clin. Pharmacol. 47, 727–737. 10.1177/0091270007300263 17519399

[B29] Wart S. A. V. Owen J. S. Ludwig E. A. Meagher A. K. Korth-Bradley J. M. Cirincione B. B. (2006). Population pharmacokinetics of tigecycline in patients with complicated intra-abdominal or skin and skin structure infections. Antimicrob. Agents Chemother. 50, 3701–3707. 10.1128/AAC.01636-05 16940069 PMC1635236

[B30] World Health Organization (2022). Global antimicrobial resistance and use surveillance system (GLASS) report 2022. Geneva: World Health Organization.

[B31] Xie J. Roberts J. A. Alobaid A. S. Roger C. Wang Y. Yang Q. (2017). Population pharmacokinetics of tigecycline in critically ill patients with severe infections. Antimicrob. Agents Chemother. 61, 003455–e417. 10.1128/AAC.00345-17 PMC5527570 28607024

[B32] Xie J. Wang T. Sun J. Chen S. Cai J. Zhang W. (2014). Optimal tigecycline dosage regimen is urgently needed: results from a pharmacokinetic/pharmacodynamic analysis of tigecycline by Monte Carlo simulation. Int. J. Infect. Dis. 18, 62–67. 10.1016/j.ijid.2013.09.008 24246741

[B33] Yaghoubi S. Zekiy A. O. Krutova M. Gholami M. Kouhsari E. Sholeh M. (2022). Tigecycline antibacterial activity, clinical effectiveness, and mechanisms and epidemiology of resistance: narrative review. Eur. J. Clin. Microbiol. Infect. Dis. 41, 1003–1022. 10.1007/s10096-020-04121-1 33403565 PMC7785128

[B34] Yang T. Mei H. Wang J. Cai Y. (2021). Therapeutic drug monitoring of tigecycline in 67 infected patients and a population pharmacokinetics/microbiological evaluation of A. Baumannii study. Front. Microbiol. 12, 678165. 10.3389/fmicb.2021.678165 34220762 PMC8241901

[B35] Yu Z. Zhao Y. Jin J. Zhu J. Yu L. Han G. (2022). Prevalence and risk factors of tigecycline-induced liver injury: a multicenter retrospective study. Int. J. Infect. Dis. 120, 59–64. 10.1016/j.ijid.2022.04.024 35429639

[B36] Zha L. Pan L. Guo J. French N. Villanueva E. V. Tefsen B. (2020). Effectiveness and safety of high dose tigecycline for the treatment of severe infections: a systematic review and meta-analysis. Adv. Ther. 37, 1049–1064. 10.1007/s12325-020-01235-y 32006240 PMC7223407

[B37] Zhen X. Lundborg C. S. Sun X. Zhu N. Gu S. Dong H. (2021). Economic burden of antibiotic resistance in China: a national level estimate for inpatients. Antimicrob. Resist. Infect. Control 10. 10.1186/s13756-020-00872-w PMC7789653 33407856

[B38] Zhou C.-C. Huang F. Zhang J.-M. Zhuang Y.-G. (2022). Population pharmacokinetics of tigecycline: a systematic review. Drug Des. Dev. Ther. 16, 1885–1896. 10.2147/DDDT.S365512 PMC9211078 35747442

[B39] Zhou Y. Xu P. Li H. Wang F. Yan H. Liang W. (2021). Population pharmacokinetics and exposure‐response analysis of tigecycline in patients with hospital‐acquired pneumonia. Br. J. Clin. Pharmacol. 87, 2838–2846. 10.1111/bcp.14692 33283892

