# Supplementary figures and images for: Population pharmacokinetics and individualized dosing of tigecycline for critically ill patients: a prospective study with intensive sampling
Source: Front Pharmacol. 2024 Jan 29;15:1342947. doi: 10.3389/fphar.2024.1342947 (PMC10859475; doi:10.3389/fphar.2024.1342947)

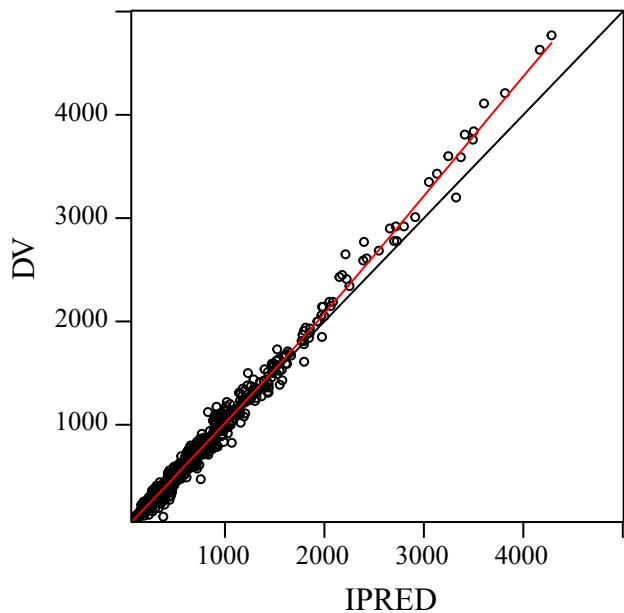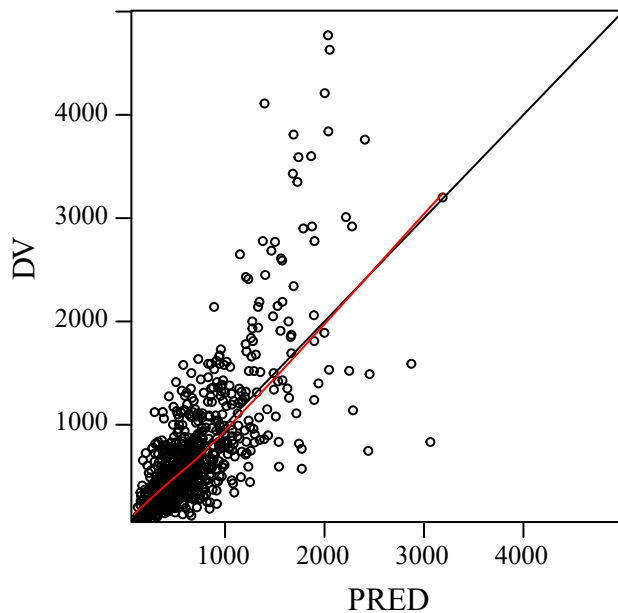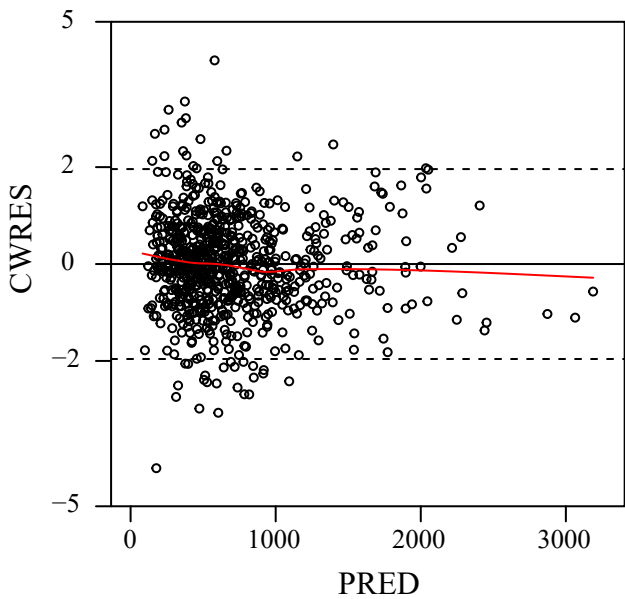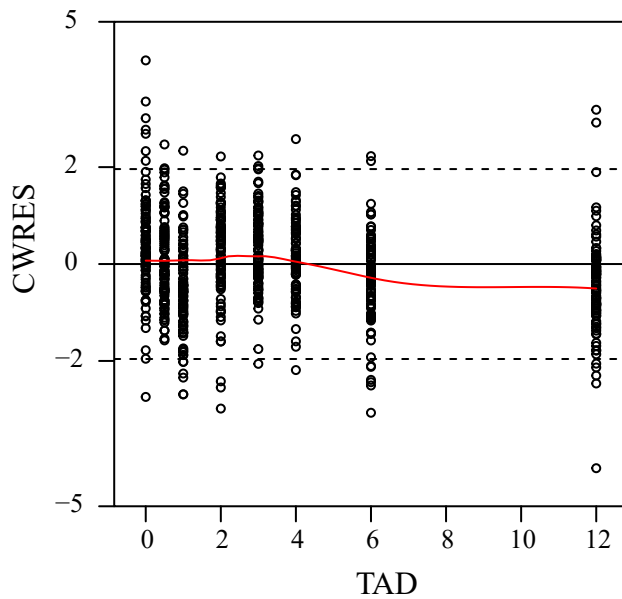

Supplement: Supplementary file 1 [file DataSheet2.PDF]

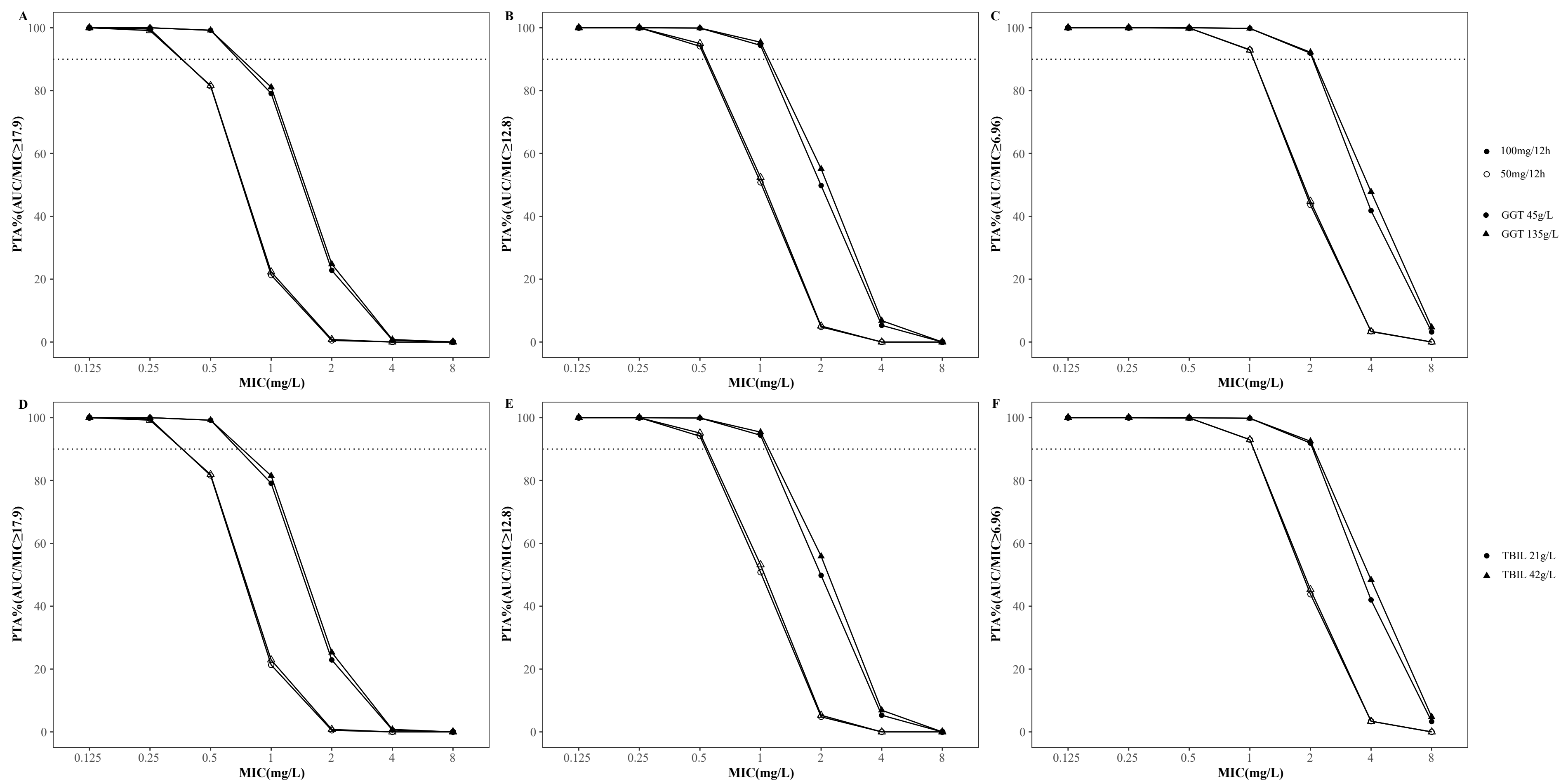

Supplement: Supplementary file 3 [file DataSheet3.PDF]

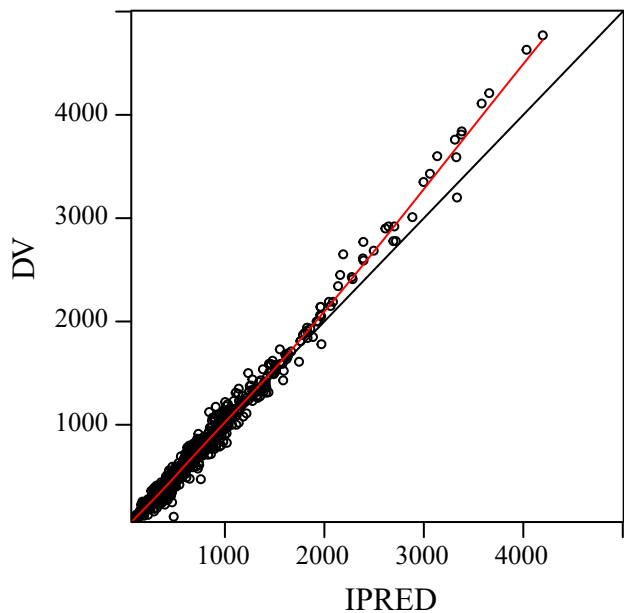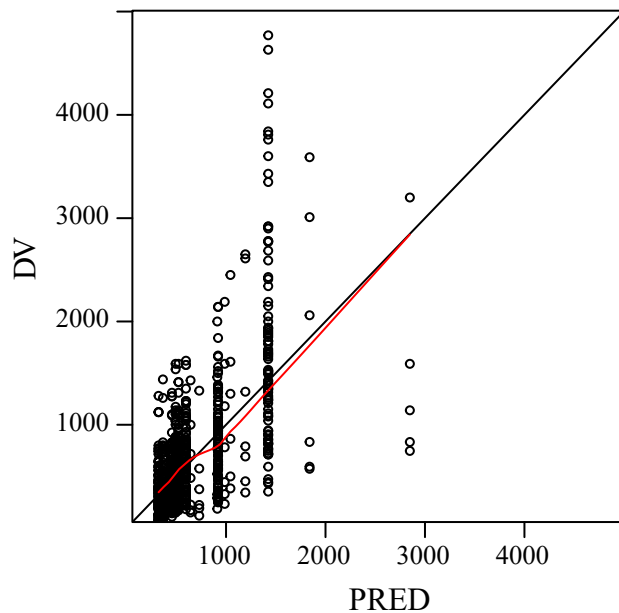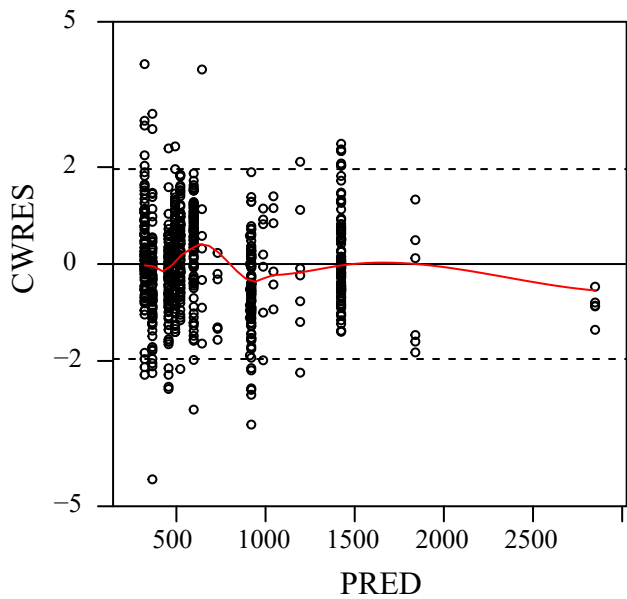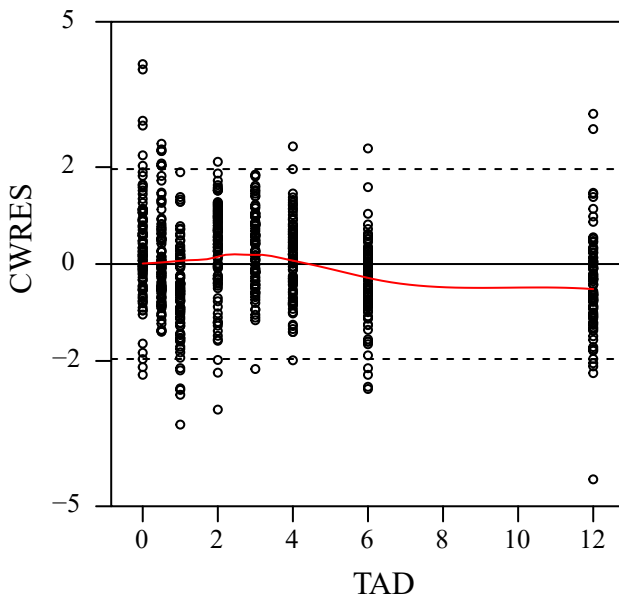

Supplement: Supplementary file 4 [file DataSheet1.PDF]
