# Supplementary material for: Population pharmacokinetics and individualized dosing of tigecycline for critically ill patients: a prospective study with intensive sampling
Source: Front Pharmacol. 2024 Jan 29;15:1342947. doi: 10.3389/fphar.2024.1342947 (PMC10859475; doi:10.3389/fphar.2024.1342947)
Supplement: Supplementary file 2 [file DataSheet4.DOCX]

Table S1 Summary of the reported pharmacokinetic studies of tigecycline.

| **Study** | **Sample size** | **Country** | **Patient group** | **Compartments** | **Covariates tested** | **Covariates included in the final model** | **CL (L/h)** | **V1 (L)** | **Q** | **V2** |
| --- | --- | --- | --- | --- | --- | --- | --- | --- | --- | --- |
| Wart et al., 2006 | 169 | America | cIAI or cSSSI | Two-compartment model | age, weight, gender, race, ALP, ALT, AST, TBIL, CCr, ALB, hematocrit,  Hb, and RBC | weight, CCr, gender | 15.7 | 115 | 70.9 | 644 |
| Rubino et al., 2010 | 410 | America | CAP or HAP | Two-compartment model | age, sex, race, height, weight, BMI, ideal body weight, BSA, ALB, CCr, APACHE II scores | BSA, CCr | 19.2 | 65.2 | 85.1 | 398 |
| Xie et al., 2017 | 10 | China | critically ill patients with severe infections | Two-compartment model | age, sex, height, body weight, BMI, BSA, SCr, CCr, ALB, APACHE II scores | BMI | 7.5 | 72.49 | NA | NA |
| Broeker et al., 2018 | 11 | German | patients with intra-abdominal infections receiving CRRT | Two-compartment model | age, sex, bilirubin, SCr, CCr | bilirubin | 18.3 | 58.7 | 56.4 | 154 |
| Moor et al., 2018 | 37 | Polish | patients with sepsis or septic shock | Two-compartment model | age, weight, height, sex, application of extracorporeal techniques (ECMO and CRRT), dialysis volume, ultrafiltration speed, extravascular lung water index, cardiac output, SOFA score, PCT | none | 22.1 | 162 |  | 87.9 |
| Zhou et al., 2021 | 89 | China | HAP | Two-compartment model | age, gender, weight, ALT, AST, SCr, TBIL, direct bilirubin, ALB | age, weight, SCr, AST | 23.1 | 105.9 | 31.9 | 124.9 |
| Yang et al., 2021 | 67 | China | HAP, cSSSI, cIAI | One-compartment model | gender, age, body weight, direct bilirubin, TBIL, ALB, ALT, AST, GGT, ALP, BUN, SCr | BUN |  |  |  |  |
| Bastida et al., 2022 | 20 | Spanish | critically ill patients with decompensated cirrhosis and severe infections | Two-compartment model | age, sex, weight, height, BMI, bilirubin, ALT, SCr, CCr, ALB, Total serum proteins, Child–Pugh, MELD score, APACHE II, SOFA score | MELD, total serum proteins | 14.8 | 63.7 | 38.4 | 233 |
| Amann et al., 2022 | 39 | German | critically ill liver-impaired patients | Two-compartment model | weight, sex, age, AST, ALT, GGT, PLT, INR, eGFR, TBIL, the maximum liver function capacity test (LiMAx test), MELD score, Child-Pugh Score | Child Pugh score, WT | 4.81-11.3 | 64.7 | 48.4 | 119 |
|  |  |  |  |  |  | MELD-score  WT | 8.57 | 64.2 | 48.7 | 119 |
|  |  |  |  |  |  | eGFR, TBIL, WT | 7.52 | 63.4 | 48 | 120 |
| Luo et al., 2023 | 54 | China | critically ill patients | One-compartment model | sex, age, weight, WBC, ALB, ALT, AST, SCr | APACHEII, age | 11.3 | 105 |  |  |

Abbreviations: CL, clearance; Q, inter-compartment clearance; V1, central volume of distribution; V2, peripheral volume of distribution; BMI, body mass index; BSA, body surface area; SCr, serum creatinine; ALT, alanine aminotransferase; AST, aspartate aminotransferase; ALP, alkaline phosphatase; GGT, γ-glutamyl transpeptadase; TBIL, total bilirubin; BUN, blood urea nitrogen; CCr, creatinine clearance; eGFR, estimated glomerular filtration rate; ALB, albumin; Hb, hemoglobin; WBC, white blood cell; RBC, red blood cell; PLT, platelet; PCT, procalcitonin; ECMO, extra-corporeal membrane oxygenation; CRRT, continuous renal replacement therapy; INR, international normalized ratio; MELD, the Model for End-Stage Liver Disease; SOFA, sequential organ failure assessment;

Table S2 Inclusion and exclusion criteria for inpatients with intermittent intravenous tigecycline.

| **Inclusion criteria**   1. Patients hospitalised in the Intensive Care Unit; 2. Empirical or targeted treatment with tigecycline is expected according to clinical needs; 3. Subjects volunteered to participate and signing informed consent. |
| --- |
| **Exclusion criteria**   1. < 18 years old; 2. History of allergy to tigecycline or tetracycline; 3. Inability to collect pharmacokinetics blood samples; 4. Insufficient demographic or clinical information; 5. The life expectancy < less than 1 month, cannot complete the course of treatment, or unable to comply with the study protocol; 6. Received intermittent haemodialysis, peritoneal dialysis or continuous renal replacement therapy.   . |

Table S3 The process of covariate screening.

| Step | Model Description | Functional form | OFV | ΔOFV | *p* value |
| --- | --- | --- | --- | --- | --- |
| Forward inclusion | |  |  |  |  |
| 1 | Base model |  | 8226.914 |  |  |
| 2 | add CCr on CLbased on 1 | linear | 8187.946 | -38.968 | < 0.001 |
| 3 | add BW on V2 based on 2 | power | 8149.837 | -38.109 | < 0.001 |
| 4 | add ALB on V2 based on 3 | power | 8133.722 | -16.115 | < 0.001 |
| 5 | add BW on V1 based on 4 | power | 8119.119 | -14.603 | < 0.001 |
| 6 | add DOSE on V1 based on 5 | power | 8108.859 | -10.26 | < 0.01 |
| 7 | add TBIL on CL based on 6 | power | 8099.713 | -9.146 | < 0.01 |
| 8 | add TBIL on Q based on 7 | power | 8091.692 | -8.021 | < 0.01 |
| 9 | add GGT on Q based on 8 | power | 8080.994 | -10.698 | < 0.01 |
| Backward elimination | |  |  |  |  |
| 10 | eliminate DOSE based on 9 |  | 8088.958 | 7.964 | > 0.001 |
| 11 | eliminate TBIL based on 10 |  | 8098.2 | 9.242 | > 0.001 |

Abbreviations: OFV, objective function value; CL, clearance; Q, inter-compartment clearance; V1, central volume of distribution; V2, peripheral volume of distribution; SCr, serum creatinine; BW, body weight; ALB, albumin; TBIL, total bilirubin; GGT, γ-glutamyl transpeptadase.
